# Community-based HCV screening: knowledge and attitudes in a high risk urban population

**DOI:** 10.1186/1471-2334-14-74

**Published:** 2014-02-10

**Authors:** Brianna L Norton, Corrine I Voils, Sarah H Timberlake, Emily J Hecker, Neela D Goswami, Kim M Huffman, Anneka Landgraf, Susanna Naggie, Jason E Stout

**Affiliations:** 1 Division of Infectious Diseases and International Health, Duke University Medical Center, Box 102358, Durham, NC 27710, USA; 2 Durham VA Medical Center and Division of General Internal Medicine, Duke University Medical Center, Durham, NC, USA; 3 School of Nursing, Duke University Medical Center, Durham, NC, USA; 4 Division of Rheumatology and Immunology, Duke University Medical Center, Durham, NC, USA

**Keywords:** Health knowledge, Attitudes, Behaviors, Healthcare disparities, Hepatitis C, Patient education, Screening

## Abstract

**Background:**

In an attempt to curtail the rising morbidity and mortality from undiagnosed HCV (hepatitis C virus) in the United States, screening guidelines have been expanded to high-risk individuals and persons born 1945–1965. Community-based screening may be one strategy in which to reach such persons; however, the acceptance of HCV testing, when many high-risk individuals may not have access to HCV specific medications, remains unknown.

**Methods:**

We set out to assess attitudes about HCV screening and knowledge about HCV disease at several community-based testing sites that serve high-risk populations. This assessment was paired with a brief HCV educational intervention, followed by post-education evaluation.

**Results:**

Participants (n = 140) were surveyed at five sites; two homeless shelters, two drug rehabilitation centers, and a women’s "drop-in" center. Personal acceptance of HCV testing was almost unanimous, and 90% of participants reported that they would still want to be tested even if they were unable to receive HCV treatment. Baseline hepatitis C knowledge was poor; however, the brief educational intervention significantly improved knowledge and increased acceptability of testing when medical access issues were explicitly stated.

**Conclusions:**

Despite inconsistencies in access to care and treatment, high-risk communities want to know their HCV status. Though baseline HCV knowledge was poor in this population, a brief on-site educational intervention improved both knowledge and acceptability of HCV testing and care. These data support the establishment of programs that utilize community-based screening, and also provide initial evidence for acceptance of the implementation of the recently expanded screening guidelines among marginalized communities.

## Background

Hepatitis C virus (HCV) is the most common blood-borne infection in the United States (US), with an estimated 4 million persons chronically infected [1]. It is the leading cause of end-stage liver disease and hepatocellular carcinoma, as well as the most common indication for liver transplantation [2,3]. Due to lack of provider, patient, and community awareness, as well as conflicting screening guidelines, this epidemic has gone largely unnoticed [2]. Consequently, 75% of persons living with HCV are unaware of their infection [4,5] and thus are at risk of developing serious sequelae of liver disease, without an opportunity for treatment and appropriate disease management. In 2007, the number of persons dying from HCV exceeded that of HIV [6], and without imminent intervention, multiple models predict a four-fold increase in morbidity and mortality from HCV over the next decade [7,8].

In an attempt to curb this epidemic and identify more people living with HCV, national screening guidelines from the Centers for Disease Control (CDC), and now the United States Preventative Task Force (USPSTF), have recently expanded to include asymptomatic individuals who belong to high-risk groups (persons injecting/ever-injected drugs, recipients of blood products or organ transplants prior to 1992, hemodialysis patients, and persons with persistently abnormal liver enzymes), as well as one-time testing in all baby-boomers (persons born between 1945–1965) [9-11]. Though these guidelines may encourage further testing within health care settings, one limitation of these screening strategies is that many persons with HCV do not have access to healthcare. Community-based screening strategies, via health departments, methadone maintenance programs, or homeless shelters may be one approach in which to reach such persons. The Department of Health and Human Services (DHHS) Viral Hepatitis Action Plan specifically calls for outreach to high-risk communities to provide opportunities to get tested, seek care, and engage in HCV education strategies [2]. The acceptance of such interventions, particularly when many high-risk individuals may not have access to HCV medications, remains unknown. We set out to assess attitudes surrounding HCV screening, as well as knowledge regarding HCV disease and treatment at several community-based testing sites that serve high-risk populations. This assessment was paired with a brief HCV educational intervention, with the aim of also evaluating post-education changes in knowledge and attitudes.

## Methods

### Setting and participants

Sites were chosen from a list of community-based HIV/sexually transmitted diseases testing sites utilized by the local public health department (Wake County Human Services). These sites- a homeless shelter, drug rehabilitation center, and drop-in center- were chosen because they serve poor, marginalized communities, of whom many are/were persons who injected drugs (PWID), thereby putting them at-risk for HCV. The S. Wilmington Street shelter is the largest homeless shelter in Raleigh, North Carolina with 234 beds, serving only men. The Raleigh Rescue Mission, another homeless shelter, serves both men and women and has 100 beds. The Healing Place (for men and women) is a recovery and rehabilitation facility for homeless people with alcohol and drug dependency. There are 180 beds at the men’s facility and 95 at the women’s facility. The Women’s Center of Wake County is a multi-service resource center that addresses issues of poverty, homelessness, and substance abuse for single women and women with families. A convenience sample of persons attending each of these settings was surveyed. The leaders of each community center advertised the study for one week prior to the event, and members chose whether to attend the program. All interviews and education took place at each site during regular hours of operation. Participation was voluntary; interest was overwhelming. The study period took place from January 2012 to May 2012. The inclusion criteria were: 1) speak/understand English; 2) age 18 or older; (3) willingness to complete a pre and post-test survey instrument and participate in a short educational intervention. The survey was anonymous and clearly labeled as to its purpose (research study), and verbal consent was performed. No identifying information was obtained from subjects, and they were compensated for participation with a five dollar grocery store gift card. The study was exempted from institutional review board review by the Duke University Medical Center Institutional Review Board.

### Study intervention

An educational intervention was given as well as pre- and post-intervention surveys. Pre- and post-intervention survey instruments were administered verbally to participants to assess knowledge and attitudes surrounding hepatitis C disease, testing, and treatment.

The survey instrument assessed socio-demographic information, access to healthcare, knowledge of HCV, and attitudes toward community HCV screening. The survey was first piloted among patients in the Duke Infectious Diseases clinic to assess question comprehension, and revisions were made accordingly. To ensure full comprehension, study investigators verbally administered the survey instruments to all participants. The post-intervention survey was performed directly after the educational intervention and consisted of a subset of the pre-intervention questions.

The educational intervention consisted of a brief (approximately 15 minute) standardized discussion of the epidemiology of HCV, clinical significance, care and treatment options, and preventative strategies, followed by a question/answer session. A spiral-bound flip-book with diagrams was used with the discussion. The intervention was designed with the assistance of professional health educators and was directed toward a fifth grade education level. The same investigator (B.N.) delivered the educational program at each site in order to maximize the consistency of the educational intervention across sites.

### Statistical analysis

The study was powered on the primary objective: assessment of screening acceptability. We chose a sample size that would permit adequate assessment of whether a majority (i.e., >50%) of participants felt that HCV screening would be acceptable in their community. Assuming that the true rate of HCV testing acceptance in the community was 60% in the underlying population, we needed 153 participants to exclude an acceptability rate of <50% with 80% power at a 5% type 1 error rate, using a one-sided significance test. The study was stopped early due to overwhelming acceptance of screening.

Continuous variables were summarized using medians/quartiles or means/standard deviations, as appropriate to the distribution. Categorical variables were summarized with frequency counts/proportions. Changes in knowledge and acceptance of HCV testing were assessed using the McNemar test to compare baseline and post-education responses. Answers of "not sure" were considered not correct (knowledge) or negative for acceptance. We created a composite knowledge score from 18 knowledge-related questions, assigning each correct answer one point. Bivariate associations between baseline knowledge and pre-selected predictor variables were assessed using t-tests with categorical variables or Pearson correlations with continuous variables. All variables significantly associated with knowledge at an alpha of <0.05 were then entered into a multivariable linear regression model, centering the age variable in the model.

## Results

### Demographics

One hundred forty participants were surveyed at 5 sites, including 2 homeless shelters, 2 drug rehabilitation centers, and a women’s "drop-in" center. The majority of participants were male (66%) and African American (57%) (Table 1). The median age was 43 years old, with a range of 18 to 62 years. Participants varied in education levels, with 16% having stopped education after elementary school. Most people had no health insurance (73%), and less than half stated they had a regular doctor (49%). Ninety-five percent of participants had heard of HCV, 56% said they knew someone with the disease, and 18% of participants stated that they had been diagnosed with HCV. Though all of these participants were surveyed at community centers serving high-risk individuals, 36% of participants still believed they were "not at all" likely to get HCV.

**Table 1:** Patient demographics

**Patient characteristics**	**% (n = 140)**
Intervention site	
Healing Place for Men (Drug Rehabilitation Center)	35%
Healing Place for Women (Drug Rehabilitation Center)	16%
Wilmington St. Homeless Shelter	21%
Women’s Center (Drop-in Center)	13%
Raleigh Rescue Mission (Homeless Shelter)	15%
Male	66%
Age (median, IQR)	46 (33,54)
Race	
White	37%
Black	57%
Other	6%
Education	
Elementary	16%
High school	39%
Some college	31%
Finished college	14%
Insurance	
None	73%
Medicaid/medicare	12%
Private	6%
VA/other	9%
Has regular doctor	49%

### Baseline attitudes

Baseline attitudes and knowledge regarding HCV and HCV screening are presented in Table 2. Baseline acceptance of community-based screening was almost universal. Ninety-seven percent of participants stated they would get a free HCV test if it were offered, with 90% reporting that they would still want to be tested even if they were *not* able to receive treatment. When told that, if positive, they would be offered free vaccines against hepatitis A and B, or lifestyle advice on how to stay healthy with HCV, additional people wanted to be tested even if treatment was not accessible to them (95% and 96% respectively).

**Table 2:** Proportions and frequencies of pre-test survey answers

**Questions (n = 140)**	**Yes**	**No**	**Unsure**
Have you heard of HCV?	95%	4%	0.7%
Do you know anyone who has HCV?	56%	39%	4%
Have you been told you have HCV?	18%	80%	2%
How likely do you think you are to get HCV?* (n = 115)			
Not at all	36%		
Somewhat	29%		
Very	10%		
Not sure	26%		
How do you think people get HCV infection?**			
Having sex?	56%	22%	22%
Shooting up (injecting) drugs?	90%	1%	9%
Using public toilets?	17%	60%	23%
Sharing supplies for snorting drugs?	54%	26%	20%
Coughing/sneezing on someone?	28%	51%	21%
Getting a homemade tattoo?	90%	2%	9%
Which of the following problems can HCV cause to your body?*			
Stroke?	11%	31%	57%
Cirrhosis/Liver failure?	81%	3%	16%
Blindness?	38%	15%	47%
Liver cancer?	62%	9%	27%
Heart attack?	18%	33%	49%
Death?	79%	3%	18%
What makes HCV worse for the people that have it?**			
Drinking coffee?	4%	56%	41%
Drinking alcohol?	82%	6%	11%
HIV infection?	82%	3%	15%
Being obese?	34%	26%	41%
Does everyone who has HCV need treatment?**	76%	10%	14%
How many people who get treated for HCV, get cured?**			
All	3%		
Some	33%		
None	29%		
Not sure	36%		
Do you think that the side effects of HCV treatment are very bad?			
Yes	21%		
Somewhat	17%		
No	14%		
Not Sure	49%		
Would you get a free blood test for HCV?	97%	2%	1%
Would you want to get treated for HCV if you tested positive?	98%	1%	1%
Would you want to be tested for HCV, if when you tested positive you could get free treatment?	99%	0%	1%
Would you still want to be tested, if you were told you could not be offered treatment?	90%	10%	0%
Would you still want to be tested if you were told you could not be offered treatment, but you could get free vaccines against HAV/HBV?	95%	5%	0%
Would you still want to be tested if you were told you could not be offered treatment, but you could get lifestyle advice on how to stay healthy with HCV?	96%	4%	1%
Do you want free HCV testing in your community?	99%	0%	1%
Do you think other people would want free HCV testing?	86%	1%	13%
If people in the community tested positive for HCV, do you think they would do the following things if they were told it would help them?			
Drink less alcohol?	39%	24%	37%
Get a shot against HAV/HBV?	83%	2%	15%
Go to the doctor for treatment?	76%	1%	22%
Do you think it will be a problem if we tested for HCV in your community but might not be able to offer treatment to everyone who’s positive?	49%	39%	12%

*Among persons not reporting HCV infection.

**Questions that were used in composite knowledge score.

Almost all participants (99%) said that they wanted free HCV testing in their community, but participants were less positive when asked if *other* people in their community would want free HCV testing in the community (86%). Almost half (49%) thought that offering community-based screening without the availability of universal treatment would be problematic. Also, only 39% of participants believed that others in their community who tested positive for HCV would drink less alcohol. But most believed that HCV positive persons would get vaccinated against hepatitis A and hepatitis B (83%) or go to the doctor for treatment (76%).

### Baseline knowledge

Baseline knowledge about HCV acquisition was variable. Although 90% of people knew that injecting drugs and getting a homemade tattoo are risk factors for HCV, 22% of people did not think sexual acquisition was possible, 17% thought HCV was transmitted from public toilets, and 28% thought HCV was transmitted from coughing or sneezing. Many people did not know what risk factors were associated with disease progression. Participants’ baseline knowledge concerning HCV treatment was also low. Sixty five percent of participants did not think HCV could be cured or did not know if it could be cured, yet 76% believed that everyone diagnosed with HCV needed to be treated. Ninety-eight percent of participants said they would want treatment if they tested positive for HCV; however, 63% did not know about treatment side effects or thought that side effects of therapy were minimal.

### Changes in knowledge and attitudes post-intervention

The brief educational intervention significantly improved knowledge about HCV (Table 3). Eighty-one (81%) percent of participants understood that treatment was not compulsory for everyone with HCV, as compared to 10% pre-education (p < 0.0001). Ninety percent (90%) of participants gave correct responses regarding HCV cure rates after the educational intervention, as opposed to 33% pre-education (p < 0.0001). After learning about the deleterious effects of alcohol in patients with HCV, significantly more participants believed that people in the community who tested positive for HCV would drink less alcohol (p = 0.003). Attitudes toward personal acceptance of HCV testing did not change after education since almost all participants wanted to be tested on the pre-intervention survey. However, the participants’ perceived acceptability of HCV screening among *other* members of the community did increase after education. Importantly, after education, participants were significantly less likely to believe that offering community-based HCV screening without guarantee of universal treatment would be problematic (49% pre-intervention believed this would be problematic vs. 35% post-education, p = 0.02).

**Table 3:** Analysis of Pre and posttest changes of answers

***Knowledge related questions***	**Pre-test**	**Post-test**	**p-value**
	**% correct**	**% correct**	**(McNemar)**
How do you think people get HCV?			
Having sex?	56%	84%	<0.0001
Shooting up (injecting) drugs?	90%	98%	0.001
Using public toilets?	60%	95%	<0.0001
Sharing supplies for snorting drugs?	54%	85%	<0.0001
Coughing/sneezing on someone?	52%	94%	<0.0001
Getting a homemade tattoo?	89%	97%	0.0074
What makes HCV worse for the people who have it?			
Drinking coffee?	45%	86%	<0.0001
Drinking alcohol?	82%	96%	<0.0001
HIV infection?	82%	89%	0.121
Being obese?	33%	80%	<0.0001
Does everyone who has HCV need treatment?	10%	81%	<0.0001
How many people who get treated for HCV get cured?	33%	90%	<0.0001
***Attitude related questions***	**Pre-test % answered yes**	**Post-test % answered yes**	**p-value (McNemar)**
Would you want to be tested for HCV, if when you tested positive you could get free treatment?	99%	100%	1.0
Would you still want to be tested, if you were told you could not be offered treatment?	90%	90%	1.0
Would you still want to be tested if you were told you could not be offered treatment, but you could get free vaccines against HAV/HBV?	95%	96%	.727
Would you still want to be tested if you were told you could not be offered treatment, but you could get lifestyle advice on how to stay healthy with HCV?	96%	95%	1.0
Do you want free HCV testing in your community?	99%	97%	0.25
Do you think other people would want free HCV testing?	86%	92%	0.077
Do you think it will be a problem if we tested for HCV in your community but might not be able to offer treatment to everyone who’s positive?	49%	35%	0.019
If people tested positive for HCV, do you think people would do the following if they were told it would help them?			
Drink less alcohol?	39%	54%	0.003
Get a shot against HAV/HBV?	83%	86%	.383
Go to the doctor for treatment?	76%	79%	.524

### Knowledge score and associations

The mean baseline knowledge score was 9.9 (SD 3.3), ranging from 0–18. Characteristics associated with greater knowledge were male gender, white race, younger age, and knowing someone with HCV (Table 4). Interestingly, participants who did not want to be tested for HCV if they were not guaranteed treatment had significantly lower knowledge scores than people who wanted to know their HCV status despite availability of treatment (p = 0.003). When dichotomizing age based on the CDC screening recommendations, participants greater than 45 years old (baby boomer generation) had mean knowledge scores that were lower than younger participants, though this did not reach statistical significance (9.5 vs. 10.4, p = 0.08). In multivariable analysis, white race, male gender, knowing a person with HCV, and wanting HCV testing even if treatment could not be offered remained associated with higher knowledge scores (Table 5).

**Table 4:** Bivariate analysis of knowledge score

**Variable (n)**	**Mean (SD) knowledge score or correlation coefficient**	**p-value**
Total	9.9 (3.3)	
Age (140)	r = -0.17	0.04
Gender		
Male (92)	10.4 (.35)	0.02
Female (48)	9.1 (.44)	
Race		
White (52)	10.8 (2.8)	
Non-white (88)	9.4 (3.5)	0.02
Regular doctor		
Yes (68)	9.8 (3.0)	0.64
No (72)	10.1 (3.6)	
Education		
Elementary (22)	9.4 (3.6)	
High school (55)	9.5 (3.4)	0.11
Some college	10.1 (3.0)	
Finished college	11.5 (2.9)	
Insurance		
Yes (102)	10.0 (3.5)	0.83
No (38)	9.8 (2.7)	
Do you know anyone with HCV?		
Yes (79)	10.5 (3.1)	0.03
No (55)	9.3 (3.5)	
Do you have HCV?		
Yes (25)	10.6 (3.8)	0.24
No (112)	9.8 (3.2)	
Would you still want to be tested if you could not get treatment?		
Yes (126)	10.2 (3.1)	0.003
No (14)	7.5 (3.7)	

**Table 5:** Multivariable analysis of characteristics associated with knowledge score

Characteristics*	**Adjusted mean**	**p-value**	**Beta coefficient (s.e.)**
Age			
31 years (-1SD below mean)	10.37	0.271	-.026 (.024)
56 years (+1SD above mean)	9.72		
Gender			
Male	10.60	0.002	-1.76 (.566)
Female	8.84		
Race			
White	10.70	0.065	-1.13 (.605)
Non-white	9.57		
Do you know someone with HCV?			
No	9.28	0.025	1.22 (.540)
Yes	10.50		
Would want to be tested even if can’t be offered treatment?			
No	7.71	0.005	2.53 (.890)
Yes	10.25		

*Referent group placed first under each category.

## Discussion

In an attempt to curtail the rising morbidity and mortality from undiagnosed HCV, the CDC and USPSTF have expanded screening guidelines to high-risk individuals and persons born 1945-1965 [9-11]. Community-based screening programs have the potential to reach such persons [12,13]; however, it is important to understand the acceptability of HCV testing in a group that may have limited access to HCV medical treatment. In this study, we found that people who access community-based, non-traditional testing sites were highly accepting of HCV screening, even without guarantee of treatment. On the other hand, high-risk individuals lacked knowledge about HCV. Nonetheless, an easy on-site educational intervention significantly improved HCV knowledge and also increased acceptability of testing.

To our knowledge, this is the first study to directly assess acceptability rates of HCV screening when access issues were explicitly stated. We found that acceptability of screening was almost universal in this population, and remained high even when participants were told that they would not be able to receive treatment. Ninety seven percent of participants said that they would personally obtain a free HCV test, and 99% stated they would want free HCV testing in their community. Even when told they would not be able to receive treatment, 90% of participants said they would still want to know their HCV status. When told that free hepatitis A/B vaccination or advice on harm reduction could be offered, almost all participants again wanted to be tested regardless of availability of medical therapy. These data demonstrate how strongly people at-risk for HCV value knowing their status.

This is important because testing persons for HCV, even without access to medication, can still be beneficial. Public health departments are in a unique position to address lack of screening and care to populations that are specifically at risk for HCV, such as the homeless, people who inject drugs (PWID), and uninsured baby-boomers [14-16]. Early HCV detection provides an opportunity for low-cost interventions that can decrease the risk of liver disease including alcohol reduction counseling, HIV testing, and immunization against hepatitis A and B that are often available through public health services [17-19]. Furthermore, knowledge of one’s disease allows opportunity to seek out health insurance (something that may become easier under the Affordable Care Act), resulting in access to therapies at an earlier stage of liver disease, which is associated with better treatment response rates and less risk of long term liver complications [20-22]. As newer medical therapies with improved efficacy and side effect profiles become increasingly available, early identification of disease by HCV screening will have greater potential to reduce poor outcomes [23-25].

Importantly, other community based organizations, such as opioid treatment centers and urban primary care clinics, have been shown to increase HCV testing rates in high prevalence populations [26-28]. These community-based settings are unique in that, not only are they able to establish HCV positivity through screening programs, they are often able to offer HCV care and medical therapy, taking advantage of an already engaged at-risk population. Through a multidisciplinary treatment model-with onsite drug, psychiatric, and medical care- HCV treatment was shown to be effective in a large population of opioid dependent patients in a methadone maintenance program in the Bronx, NY [29]. Community-based screening can therefore act as the initial step to improving the entire cascade of HCV care for hard-to-reach populations.

Though acceptance of HCV screening in our high-risk population was high, knowledge regarding HCV was relatively poor. This lack of knowledge was surprising given over half of participants reported knowing someone with HCV and 18% of participants stated that they personally were infected with HCV. Our work supports prior investigations that have shown significant gaps in HCV knowledge in high-risk groups, such as persons living with HIV and intravenous drug users [30-33]. Similar to these studies, we found that lack of knowledge was associated with African American race, a group that is disproportionately afflicted by this disease. Participants demonstrated poor knowledge about HCV acquisition, which may impact a person’s ability to make choices that protect themselves and prevent transmission to others in their community. As shown previously [34], we also found a large percentage of people who did not know that alcohol or HIV could worsen HCV disease progression, and even fewer knew that obesity has a negative effect on liver health. This dearth of information makes it difficult for HCV positive people to make healthy lifestyle choices when living with HCV. There were also significant misconceptions in understanding HCV therapy, as most people believed that it was necessary to treat all HCV positive persons. A majority of participants were also unsure or did not think that HCV could be cured and over half carried erroneous beliefs regarding HCV treatment side effects. This supports previous work by Krauskopf et al., where only 25% of an inner-city community believed there to be a cure for HCV [33]. This hinders the ability of HCV positive persons to appropriately interpret their disease and lessens their interest in care, potentially contributing to the persistently low uptake of HCV treatment [35,36].

Fortunately, a brief educational intervention significantly improved HCV knowledge among the participants. Almost all areas of HCV knowledge improved post-intervention, with the greatest changes occurring in understanding of treatment. Notably, improvement in HCV knowledge has been shown to improve compliance with linkage to HCV care [35,36].

By increasing knowledge, our educational tool also increased acceptance rates for HCV testing. Although personal acceptance of testing was high at baseline, some participants expressed concern regarding the community’s desire for screening if access to treatment was not universal. That said, those who were concerned about community acceptance demonstrated significantly more positive attitudes toward HCV screening after education was provided. Furthermore, the small minority of individuals hesitant to be personally tested without a guarantee of treatment demonstrated lower HCV knowledge scores, even when adjusted for other variables. This is consistent with other studies that show improved knowledge leads to greater interest in HCV care [36-38]. These findings underscore the importance of continued community education to enhance both knowledge of HCV and acceptance of HCV screening and care.

This study has several limitations. Our population consisted of a convenience sample of high-risk individuals that access non-traditional testing sites of an urban health department in the southern United States. Though this is a highly specific community, these participants did indeed have traditional risk factors for HCV that one would expect in other at-risk populations, such as drug use, homelessness, and African American race. Because many of the participants were in drug/alcohol rehabilitation programs, they may have been more motivated to provide positive responses to survey questions than others in their communities. Verbal administration of the surveys may have also biased the participants to provide more positive responses than a written instrument. Therefore, our results regarding acceptance may not be generalizable to the entire at-risk population. Also, we assessed the impact of the educational intervention immediately following the discussion, so we cannot comment as to whether the improvement in knowledge was durable, though other studies of brief educational interventions have shown sustainability of knowledge from 1 to 18 months later [39,40]. Finally, since a member of the study team verbally administered the education intervention, its reproducibility cannot be guaranteed.

## Conclusions

Acceptance of community-based HCV screening amongst a high-risk population was almost universal, even without guarantee of treatment. Despite inconsistencies in availability of HCV medications and poor knowledge regarding HCV, high-risk communities are ready to know their HCV status. Furthermore, a screening strategy that implements brief on-site education can aid in improving HCV knowledge and engagement in care and testing. These data support the establishment of programs that utilize community-based screening, and also provide initial evidence for acceptance of the implementation of the recently expanded screening guidelines among marginalized communities.

## Competing interests

The authors declare that they have no competing interests.

## Authors’ contributions

BN took part in the conception, design, acquisition of data, data analysis, and was the primary person in drafting the manuscript. CV took part in the conception, design, analysis and interpretation of data, as well as revising the manuscript. ST took part in the acquisition of data, analysis of data and revising manuscript. EH took part in the design of the project, acquisition of data, and revision of manuscript. NG took part in the design of the project, acquisition of data, and revision of manuscript. KH took part in the acquisition of data, analysis and interpretation of data, and revision of manuscript. AL took part in the acquisition of data, and revision of manuscript. SN took part in the conception and design of project, interpretation of data, and revision of manuscript. JS took part in the conception and design of the project, acquisition of data, data analysis, and revision of the manuscript. All authors read and approved the final manuscript.

## Pre-publication history

The pre-publication history for this paper can be accessed here:

http://www.biomedcentral.com/1471-2334/14/74/prepub
